# Anthocyanin-Rich Freeze-Dried Berry Supplementation in Patients Undergoing Colorectal Cancer Treatment: A Prospective Single-Arm Pilot Study of Tolerability and Longitudinal Inflammatory Biomarker Changes

**DOI:** 10.3390/nu18213571

**Published:** 2026-10-29

**Authors:** Katarzyna Jaglarz-Biały, Marcin Surmiak, Mirosław Krośniak, Mirosława Püsküllüoğlu, Aleksander Konturek

**Affiliations:** 1 Doctoral School of Medical and Health Sciences, Jagiellonian University Medical College, 31-530 Krakow, Poland; 2 Department of Clinical Oncology, St Luke’s Hospital, 33-100 Tarnow, Poland; 3 Bioengineering and Cell Imaging Laboratory, Center for the Development of Therapies for Civilization and Age-Related Diseases, Jagiellonian University Medical College, 31-066 Krakow, Poland; 4 Department of Food Chemistry and Nutrition, Jagiellonian University Medical College, 30-688 Krakow, Poland; 5 Department of Clinical Oncology, Maria Sklodowska-Curie National Research Institute of Oncology, Cracow Branch, Garncarska 11, 31-115 Krakow, Poland; 6 Department of Endocrine Surgery, Jagiellonian University Medical College, 31-501 Krakow, Poland

**Keywords:** anthocyanins, colorectal cancer, systemic inflammation, inflammatory biomarkers, *C*-reactive protein, supportive care, functional food

## Abstract

**Background/Objectives:** Anthocyanin-rich foods and supplements have shown anti-inflammatory activity in experimental studies, but clinical evidence in patients with colorectal cancer (CRC) remains limited. This exploratory pilot study evaluated longitudinal changes in systemic inflammatory biomarkers and the feasibility and tolerability of anthocyanin-rich berry supplementation during CRC treatment. **Methods:** In this prospective, open-label interventional study, 50 patients with CRC received an anthocyanin-rich freeze-dried whole-berry supplement (estimated anthocyanin intake of approximately 600–700 mg/day) for 21 consecutive days before initiation of oncological treatment and were followed for 12 weeks. Twenty non-cancer individuals served exclusively as a baseline comparator group. The comparator group was used only for baseline comparisons and not for longitudinal assessment of treatment effects. Clinical and laboratory assessments were performed at baseline, Week 4 (before initiation of oncological treatment), and Week 12 (during standard oncological treatment). Longitudinal changes in inflammatory and biochemical biomarkers were analyzed using non-parametric methods. **Results:** Median CRP concentrations decreased significantly during follow-up (*n* = 43), from 20.4 mg/L at baseline to 5.2 mg/L at Week 4 (following supplementation but before initiation of oncological treatment), and to 3.0 mg/L at Week 12 (during standard oncological treatment, *p* < 0.001). Significant longitudinal changes were also observed in interleukin-1α (IL-1α) net median fluorescence intensity (Net MFI), tumour necrosis factor-α (TNF-α) Net MFI, and interleukin-6 (IL-6) (*n* = 39). The supplement was generally well-tolerated among participants with available tolerability data (*n* = 47). **Conclusions:** A 21-day course of anthocyanin-rich berry supplementation was generally well-tolerated in patients with colorectal cancer. Longitudinal changes in systemic inflammatory biomarkers were observed during follow-up. Because the study did not include a longitudinal CRC control group and participants continued standard oncological treatment, these findings should be considered exploratory and cannot establish that the observed biomarker changes were caused by the supplementation. The observed biomarker patterns are hypothesis-generating and warrant confirmation in adequately powered controlled studies to determine whether anthocyanin-rich berry supplementation influences systemic inflammatory biomarkers in patients with CRC.

## 1. Introduction

Colorectal cancer (CRC) is a major global health challenge and remains among the most frequently diagnosed malignancies and leading causes of cancer-related deaths worldwide. More than 1.9 million new CRC cases and approximately 904,000 deaths were estimated worldwide in 2022 [1]. The burden of CRC is particularly high in developed regions, including Europe, emphasizing the continued need for effective preventive and supportive strategies. Although genetic alterations represent fundamental drivers of colorectal carcinogenesis, increasing evidence indicates that metabolic disturbances, dietary factors, oxidative stress, and chronic inflammation play important roles in tumour initiation and progression [2,3].

Dietary patterns characteristic of highly industrialized countries, particularly the Western diet rich in processed red meat, saturated animal fats, and refined carbohydrates and low in dietary fiber, have been associated with increased CRC risk. Conversely, epidemiological studies have demonstrated an inverse association between fruit and vegetable consumption and CRC risk, suggesting a potential role for plant-derived bioactive compounds in colorectal cancer prevention [4].

Chronic inflammation and oxidative stress are important biological pathways linking diet with colorectal carcinogenesis, contributing to epithelial damage and alterations in the tumour microenvironment [5,6]. Systemic inflammation also has prognostic relevance in CRC; higher postdiagnosis levels of inflammatory biomarkers have been associated with increased risks of recurrence and mortality, although reductions in individual biomarkers should not be interpreted as surrogate evidence of clinical benefit [7].

Plant-derived bioactive compounds, particularly polyphenols and other secondary metabolites, have long attracted interest in oncology because of their diverse biological activities and their established role as a source of anticancer agents [8]. Anthocyanins are flavonoid polyphenols with anti-inflammatory and antioxidant properties. Experimental studies indicate that anthocyanins may modulate oxidative stress and inflammatory signaling, including the production of pro-inflammatory mediators such as TNF-α, IL-1β, and IL-6 [9,10,11].

Although experimental studies support anti-inflammatory effects of anthocyanins, evidence from human interventions is less consistent. Meta-analyses of randomized trials suggest modest reductions in selected inflammatory biomarkers, including CRP, IL-6, and TNF-α, with substantial heterogeneity according to population, formulation, dose, and intervention duration [12,13]. Clinical evidence specifically in colorectal neoplasia remains limited. Short presurgical studies using standardized bilberry anthocyanin extracts or freeze-dried black raspberries have demonstrated tissue exposure and changes in biomarkers related to proliferation, apoptosis, Wnt signaling, and angiogenesis in patients with CRC [14,15]. However, effects on circulating inflammatory biomarkers have been less consistent [16]. Similarly, a randomized presurgical study in patients with colorectal adenomas found no significant between-group differences in changes in circulating inflammatory biomarkers following combined anthocyanin and curcumin supplementation [17]. Clinical experience with anthocyanin-rich preparations has also been reported in other oncological settings. In patients with oral squamous cell carcinoma, short-term administration of freeze-dried black raspberry troches resulted in detectable berry-derived phytochemicals in tumour tissue and changes in pro-inflammatory and pro-survival molecular biomarkers [18]. Conversely, in a randomized placebo-controlled trial involving patients with breast cancer undergoing radiotherapy, anthocyanin-rich supplementation was well-tolerated but did not prevent radiation-induced skin toxicity [19].

Although these findings provide preliminary clinical evidence for anthocyanin-rich interventions, available studies have predominantly involved small presurgical or chemoprevention settings rather than patients undergoing routine treatment for established CRC. Importantly, evidence of preventive or tumour-related biomarker effects in presurgical settings should be distinguished from the modulation of systemic inflammatory biomarkers in patients with established CRC undergoing oncological treatment. Evidence on the tolerability of anthocyanin-rich supplementation and longitudinal systemic inflammatory biomarker patterns during routine CRC treatment therefore remains particularly limited. In particular, little is known about longitudinal changes in systemic inflammatory biomarkers following anthocyanin-rich supplementation in older patients with CRC, a population characterized by increased inflammatory burden, immunosenescence, higher prevalence of nutritional impairment, and greater vulnerability to treatment-related complications. Furthermore, the optimal dose and clinical relevance of anthocyanin supplementation in this population remain insufficiently established.

Therefore, the aim of this prospective interventional pilot study was to characterize longitudinal changes in systemic inflammatory and biochemical biomarkers in patients with colorectal cancer receiving anthocyanin-rich berry supplementation before standard oncological treatment. Additionally, the study assessed the feasibility and tolerability of the supplementation during colorectal cancer treatment.

## 2. Materials and Methods

### 2.1. Study Design and Participants

This prospective, open-label, single-arm interventional pilot study with a baseline non-cancer comparator group was designed to evaluate longitudinal changes in a panel of inflammatory and biochemical biomarkers and the feasibility and tolerability of oral anthocyanin-rich supplementation in patients with colorectal cancer. The biomarker assessment was intentionally based on a panel of inflammatory and biochemical markers rather than on a single formally designated primary outcome. Accordingly, no individual biomarker was designated as the sole primary endpoint, and the individual biomarker analyses presented in this study should be regarded as exploratory and hypothesis-generating. Within the biomarker panel, CRP was of particular clinical interest because it is a routinely available inflammatory marker that can be repeatedly assessed in everyday oncology practice without the need for specialized analytical methods. As this was an exploratory pilot study, no formal a priori power calculation was performed. The sample size was feasibility-based and was determined by recruitment feasibility within the study timeframe and by the available resources and assay capacity for multiplex biomarker analyses. In particular, the requirement to identify and enroll eligible patients before initiation of oncological treatment substantially restricted recruitment.

The study was conducted in accordance with the Declaration of Helsinki and was approved by the Bioethics Committee of the Jagiellonian University (protocol code 1072.6120.80.2022, approved on 15 March 2023). All participants provided written informed consent prior to enrolment. The study was conducted as an independent academic initiative without industry support or commercial funding. The study was retrospectively registered at ClinicalTrials.gov (NCT07819539).

Between May 2023 and December 2025, consecutive patients with histologically confirmed colorectal adenocarcinoma were recruited during their first outpatient visit to the Departments of Medical Oncology, Radiation Oncology, and Surgical Oncology at the Tarnów Oncology Centre. Patients were evaluated before initiation of standard oncological treatment and had been qualified for surgery, neoadjuvant chemoradiotherapy, or systemic therapy.

Overall, 70 participants were enrolled in the study. The intervention group comprised 50 patients with histologically confirmed colorectal adenocarcinoma who subsequently received anthocyanin supplementation. An additional 20 individuals without colorectal cancer who were undergoing colonoscopy constituted a baseline comparator group. The comparator group was included exclusively to provide a reference for baseline demographic, clinical, and laboratory characteristics and did not participate in the longitudinal intervention phase.

The inclusion criteria for the intervention group were: (1) histologically confirmed primary colorectal adenocarcinoma; (2) qualification for surgical treatment, neoadjuvant chemoradiotherapy, or systemic chemotherapy; (3) ability to receive oral supplementation; (4) Eastern Cooperative Oncology Group (ECOG) performance status ≤ 2. For the purposes of the original study protocol, older age was defined a priori as ≥65 years. However, six participants aged 61–64 years who otherwise fulfilled the eligibility criteria were also enrolled, representing a deviation from the prespecified age criterion.

The exclusion criteria for the intervention group comprised: (1) gastrointestinal obstruction; (2) advanced cardiac or respiratory failure; (3) severe renal or hepatic insufficiency; (4) food allergies or hypersensitivity that could interfere with administration of the study supplement.

**Figure 1:**
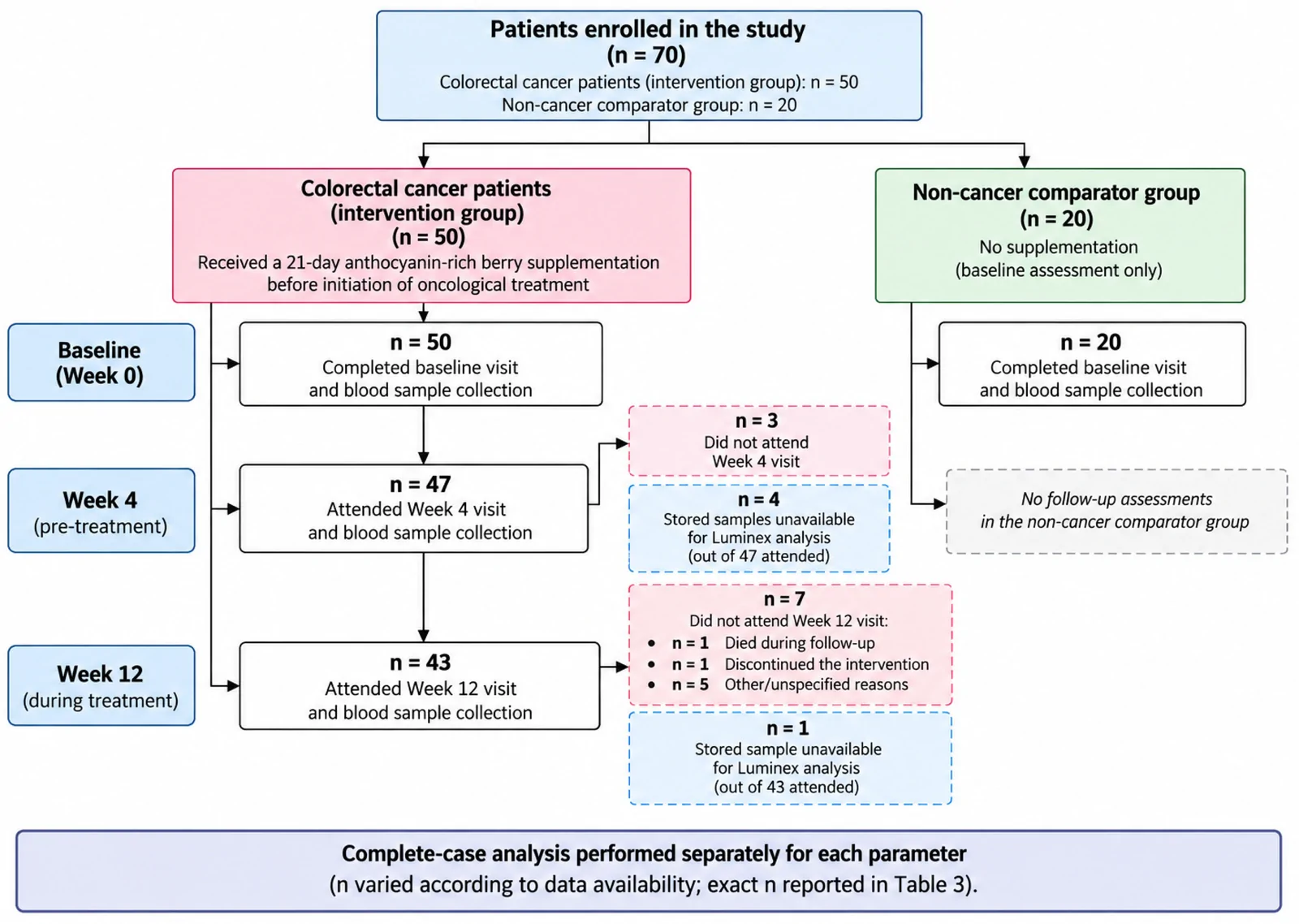
Study flow chart. Longitudinal analyses included participants with complete data at all three time points for each respective parameter; therefore, sample sizes varied according to data completeness.

The study flow diagram is presented in Figure 1.

### 2.2. Study Procedures

At baseline (Week 0), all participants underwent a comprehensive clinical evaluation. Demographic and clinical data, including comorbidities and concomitant medications, were recorded. Performance status was assessed according to the ECOG scale. Anthropometric measurements included body weight, height, and body mass index (BMI). Nutritional status was evaluated using the Mini Nutritional Assessment (MNA) and the Mini Nutritional Assessment–Short Form (MNA-SF). Peripheral venous blood samples were collected after enrolment for the assessment of *C*-reactive protein (CRP), total protein, albumin, interleukin (IL)-1α, IL-1β, IL-6, IL-8, IL-10, tumour necrosis factor-α (TNF-α), vascular endothelial growth factor (VEGF), epidermal growth factor (EGF), and carcinoembryonic antigen (CEA). The same baseline assessment protocol was applied to participants in the baseline comparator group. Peripheral venous blood samples were collected from fasting participants into standard serum tubes used for routine biochemical testing. After collection, blood was allowed to clot for at least 30 min and was centrifuged on the same day at 4000 rpm for 10 min, according to the standard operating procedure of the hospital laboratory. Following serum separation, each sample was aliquoted into four Eppendorf tubes and stored frozen at −22 °C until analysis. Samples were analysed in batches after a sufficient number of specimens had been accumulated for a Luminex assay run. For multiplex analysis, one aliquot was thawed and used for the assay, while the remaining aliquots were retained frozen as backup material. Thus, the aliquot used for Luminex analysis was not subjected to repeated freeze–thaw cycles.

The 21-day supplementation intervention was administered before initiation of the planned oncological treatment. Following the baseline evaluation, participants in the supplementation group received, free of charge, an oral anthocyanin-rich freeze-dried whole-fruit preparation developed specifically for this study by the Department of Bromatology, Faculty of Pharmacy, Jagiellonian University Medical College. The intervention consisted of compressed freeze-dried whole-fruit material from black raspberry and strawberry rather than a purified anthocyanin compound; therefore, anthocyanin exposure is reported as an approximate range rather than as a single fixed dose. In addition to anthocyanins, the formulation contained other biologically active phytochemicals. Previously published analyses describe the phytochemical composition and antioxidant properties of selected freeze-dried fruit materials relevant to the formulation used in the present study [20]. Participants received oral supplementation for 21 consecutive days at a dose of five tablets three times daily, corresponding to an estimated daily anthocyanin intake of approximately 600–700 mg (reflecting the natural variability in the anthocyanin content of the preparation). This estimate was not verified by batch-specific quantitative analysis of the tablets administered in the present study. No study supplementation was administered after Day 21. Participants continued their habitual diet throughout the study. No standardized dietary regimen or specific dietary restrictions were imposed, and dietary intake, including consumption of foods naturally rich in anthocyanins or other polyphenols, was not systematically controlled. The 21-day intervention period was selected to allow completion of the supplementation before initiation of the planned oncological treatment, without delaying standard cancer therapy.

### 2.3. Follow-Up Assessments

The first follow-up assessment was performed four weeks after baseline. Both the baseline assessment and the Week 4 follow-up assessment were performed before treatment initiation. Standard oncological treatment was initiated thereafter, and the Week 12 assessment was therefore performed during ongoing oncological treatment. Treatment was selected according to disease stage and routine clinical indications. In patients requiring systemic therapy, treatment selection was based on contemporary clinical guidelines, including ESMO and NCCN recommendations, taking into account disease stage and relevant predictive factors. The clinical evaluation, anthropometric measurements, ECOG performance status assessment, and nutritional screening (MNA and MNA-SF) were repeated. Post-supplementation adherence was assessed by patient self-report. Participants were asked about the completion of the 21-day supplementation period and about the practical tolerability of the formulation, including taste, swallowing difficulties, discomfort related to fruit seeds, and excessive tablet burden. Adherence was not independently verified by systematic pill counts or another objective adherence measure. Tolerability was assessed qualitatively based on participant report during the Week 4 clinical interview; no validated tolerability questionnaire or formal adverse-event grading scale was used. Peripheral venous blood samples were obtained for repeated measurement of CRP, total protein, albumin, IL-1α, IL-1β, IL-6, IL-8, IL-10, TNF-α, VEGF, EGF, and CEA. The final follow-up visit was performed 3 months (12 weeks) after enrolment. The same clinical, nutritional, anthropometric, and laboratory assessments were repeated according to the study protocol. Statistical analyses were performed using all collected clinical, anthropometric, nutritional, and laboratory data. Baseline characteristics of the supplementation and baseline comparator groups were compared to assess their comparability before the intervention. Longitudinal analyses were subsequently performed to evaluate changes in inflammatory, nutritional, angiogenic, and tumour-related biomarkers within the supplementation group at baseline, Week 4, and Week 12. Changes in nutritional status and anthropometric parameters over the study period were also analysed.

### 2.4. Evaluation of Serum Proteins

Serum levels of cytokines and growth factors, including interleukin (IL)-1α, IL-1β, IL-6, IL-8, IL-10, tumour necrosis factor-α (TNF-α), and vascular endothelial growth factor (VEGF), were measured using a magnetic bead-based Luminex xMAP assay (custom-made kit, Bio-Techne, Minneapolis, MN, USA) on the xMAP INTELLIFLEX^®^ System (Luminex, Austin, TX, USA). All analytes were measured simultaneously within the same multiplex assay. Concentrations of IL-6, IL-8, IL-10, and VEGF were calculated from the corresponding standard curves and are reported in pg/mL. For IL-1α, IL-1β, and TNF-α, however, the fluorescence signals in the analysed samples were below the concentration range defined by the lowest standards provided in the assay (4.7 pg/mL for IL-1α, 18.23 pg/mL for IL-1β, and 7.4 pg/mL for TNF-α, respectively). Therefore, reliable quantitative concentrations could not be calculated for these analytes. To retain potentially biologically relevant differences in signal intensity between samples, the IL-1α, IL-1β, and TNF-α results are presented as net median fluorescence intensity (Net MFI) rather than as calculated concentrations. Net MFI was calculated by subtracting the median fluorescence intensity of the blank from that of the respective serum sample. The blank contained all assay reagents, with manufacturer-provided buffer used instead of serum, and therefore represented background fluorescence. Net MFI values were used exclusively for relative comparisons between samples and were not interpreted as absolute analyte concentrations. Accordingly, longitudinal differences in IL-1α, IL-1β, and TNF-α Net MFI should not be interpreted as quantitative changes in circulating cytokine concentrations. Serum *C*-reactive protein (CRP), total protein, albumin, and carcinoembryonic antigen (CEA) levels were assessed as part of routine laboratory testing in cancer patients and were measured by the Clinical Chemistry Laboratory of the Tarnów Oncology Centre.

### 2.5. Statistical Analysis

Mean, standard deviation, median, quartiles and range of quantitative variables were shown. For qualitative variables, absolute and relative frequencies (*N* and %) were reported. Qualitative variables were compared between groups using the chi-squared test or Fisher’s exact test, as appropriate. Mann–Whitney test was used for comparisons of quantitative variables between two groups, while Kruskal–Wallis test (followed by post hoc Dunn test) was used for three or more groups. Spearman’s correlation coefficient was used to assess correlation between two quantitative variables. Friedman test was used to compare three repeated measures of quantitative variables. Longitudinal analyses across all three time points (baseline, Week 4, and Week 12) were performed using the Friedman test and included only participants with complete data at all three assessments. Complete-case eligibility was determined separately for each parameter; therefore, sample sizes differed between longitudinal analyses. Analyses of changes between baseline and Week 12 were based on participants with paired measurements available at these two time points; therefore, sample sizes differed between analyses. Paired Wilcoxon tests with Bonferroni correction were used for post hoc pairwise comparisons between time points within each biomarker following the Friedman test. No additional multiplicity adjustment was applied across the biomarker panel, given the exploratory, hypothesis-generating nature of this pilot study. Accordingly, the biomarker-specific findings should be interpreted as exploratory rather than confirmatory. Where applicable, effect-size estimates with 95% confidence intervals were reported for overall analyses. Significance level was set to *p* < 0.05. All the analyses were conducted in R software, version 4.6.1.

## 3. Results

Of the 50 patients in the intervention cohort, 47 attended the Week 4 assessment and 43 attended the complete Week 12 assessment. The number of available measurements differed between individual parameters because of missed visits and unavailable laboratory samples. Longitudinal analyses using the Friedman test were performed separately for each parameter and included participants with complete measurements for that specific parameter at baseline, Week 4, and Week 12. Consequently, the number of complete cases differed between analyses and is reported separately for each parameter. Clinical data available from routine oncological care, including anthropometric measurements, treatment information, and performance status, were retained where available. The baseline characteristics of the intervention and baseline comparator groups are presented in Table 1. Regarding disease stage, 11 patients (22%) had stage II, 23 (46%) had stage III, and 16 (32%) had stage IV disease. Oncological management during the study period was heterogeneous: 17 patients (34%) underwent surgery as the principal treatment strategy, 14 (28%) received postoperative adjuvant systemic therapy, 3 (6%) received preoperative chemoradiotherapy, and 16 (32%) received systemic treatment for advanced/metastatic disease. Importantly, the Week 4 assessment was performed before initiation of oncological treatment, whereas the Week 12 assessment was performed during the respective treatment pathways. Among demographic and clinical characteristics, the only statistically significant difference between the groups was sex distribution, with a higher proportion of women in the baseline comparator group. At baseline, the intervention group had higher CRP and CEA concentrations and higher IL-1β Net MFI values than the baseline comparator group (Table 2). No statistically significant between-group differences were observed for the remaining laboratory parameters.

Although changes in body weight, body mass index (BMI), the albumin-to-CRP ratio, and nutritional status assessed by the Mini Nutritional Assessment (MNA) and MNA-Short Form (MNA-SF) were evaluated during the study, these findings will be reported separately. The present manuscript focuses on longitudinal changes in biochemical and inflammatory biomarkers, with particular emphasis on CRP and circulating cytokine-related measures.

Longitudinal analysis demonstrated significant changes in several biomarkers during follow-up. CRP concentrations changed significantly over the 12-week follow-up (*n* = 43, *p* < 0.001), with median concentrations decreasing from 20.44 mg/L (IQR 2.84–46.38) at baseline to 5.22 mg/L (IQR 2.03–36.02) at Week 4 and 3.00 mg/L (IQR 1.27–15.84) at Week 12. The overall effect size was Kendall’s W = 0.24 (95% CI 0.07–0.52). Post hoc comparisons showed significant differences between baseline and Week 4 and between Week 4 and Week 12. IL-1α Net MFI also changed significantly over time (*n* = 39, *p* = 0.003), with lower values at Week 12 than at baseline and Week 4. TNF-α Net MFI was lower at Week 12 than at baseline (*n* = 39; overall *p* < 0.001). IL-6 concentrations differed across the three assessments (*n* = 39, *p* = 0.038), with the only significant post hoc difference observed between Week 4 and Week 12. No significant longitudinal changes were observed for IL-1β Net MFI, IL-10, IL-8, VEGF, EGF, CEA, total protein, or albumin (Table 3).

**Figure 2:**
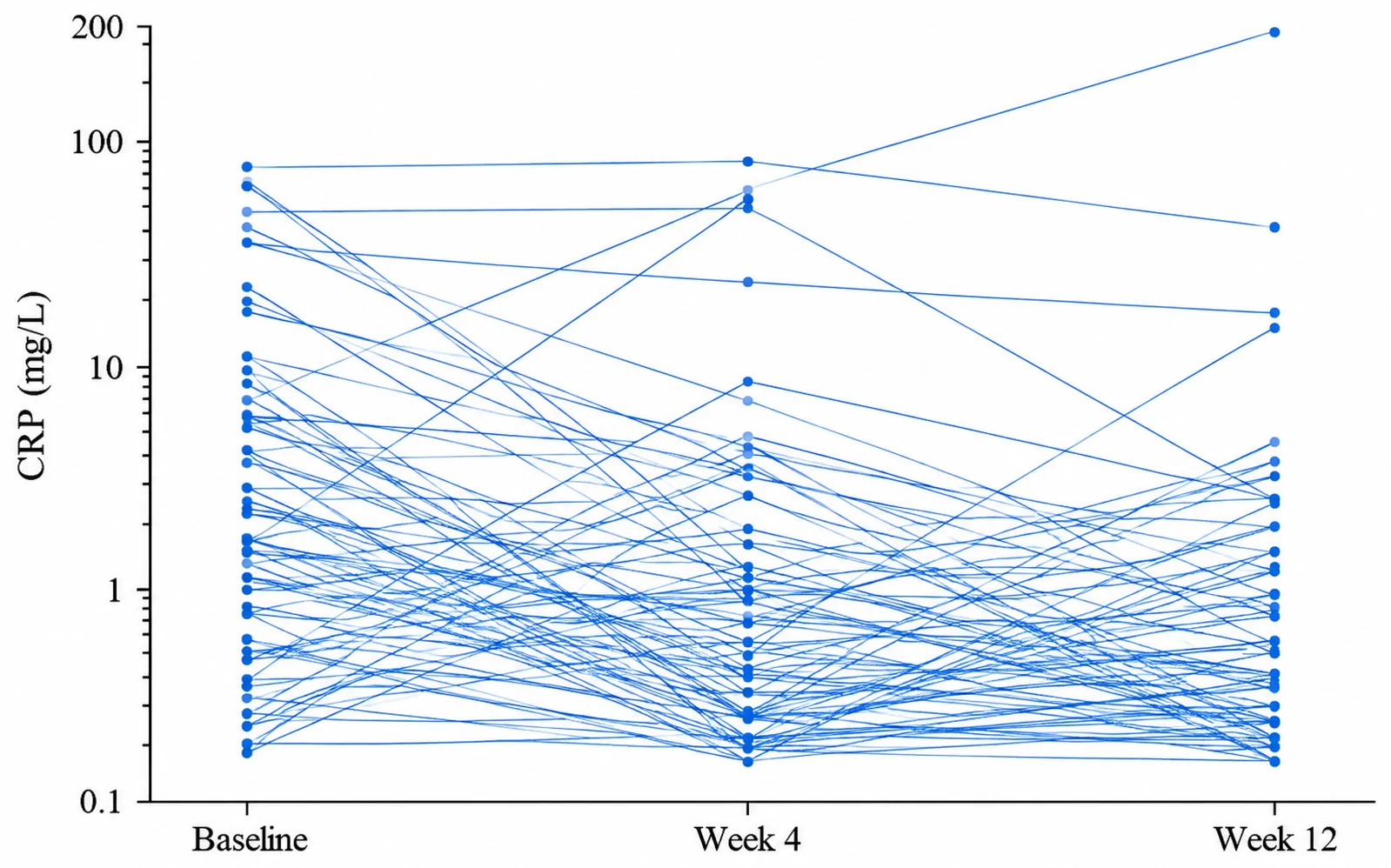
Individual longitudinal trajectories of *C*-reactive protein (CRP) from baseline to Week 12. Each line represents one participant with complete CRP measurements at all three time points. CRP values are presented on a logarithmic scale to accommodate the skewed distribution and wide range of values.

The median reduction in CRP concentration after 3 months was 11.9 mg/L (IQR 0–34.3). Overall, CRP concentrations decreased in 72.1% of patients, with reductions of at least 20% and 50% observed in 69.8% and 53.5% of patients, respectively. These thresholds were used solely as descriptive exploratory categories and were not prespecified clinical response criteria. More than half of the patients with baseline CRP concentrations between 5 and 50 mg/L achieved CRP levels below 5 mg/L after 3 months. Patients with higher baseline CRP concentrations experienced greater reductions over time, as demonstrated by a significant positive correlation between baseline CRP concentration and the magnitude of CRP reduction (Spearman’s ρ = 0.80, *p* < 0.001; Table 4 and Table 5, Figure 2).

Although IL-8 concentrations did not change significantly over the three assessment time points, IL-8 concentrations at Week 12 were lower than baseline in 52.4% of patients, including reductions of at least 20% in 45.2% and at least 50% in 28.6% of patients (Table 6 and Table 7). Similarly, VEGF concentrations decreased in 64.3% of patients, with reductions of at least 20% and 50% observed in 42.9% and 14.3% of patients, respectively. In both cases, higher baseline concentrations were associated with greater reductions after 3 months of follow-up (Table 8 and Table 9).

At the Week 4 assessment, participants with available adherence data who had not discontinued the intervention reported completion of the supplied 21-day supplementation course; adherence was based on self-report and was not independently verified by tablet counts. Tolerability data were available for 47 participants, of whom 37 (78.7%) rated tolerability as good (Table 10). Complaint-specific data were available for 48 participants; for two participants, information on specific complaints was unavailable. One participant discontinued the intervention before completing the supplementation course and returned the unused tablets. The most frequently reported problems involved the presence of small fruit seeds in the freeze-dried formulation, while two participants (4%) considered the daily tablet burden excessive (Table 11). Despite these limitations, overall acceptability of the supplement was good, with participants reporting favorable palatability and ease of incorporation into commonly consumed foods and beverages such as yogurt, milk, and tea. In an exploratory analysis, participants reporting medium/low tolerability were older than those reporting good tolerability (*p* = 0.005). The corresponding effect size was η^2^ = 0.193 (95% CI 0.053–0.361). No significant differences in tolerability were observed according to baseline BMI, MNA, MNA-SF, or ECOG performance status.

## 4. Discussion

In this prospective single-arm pilot study, the most clinically interpretable longitudinal finding was a significant decrease in CRP during follow-up, with an early reduction already evident at Week 4 before initiation of oncological treatment. Additional exploratory longitudinal changes were observed in IL-1α Net MFI, TNF-α Net MFI, and IL-6. Together, these findings describe a broader inflammatory biomarker pattern in patients who received anthocyanin-rich berry supplementation, but they should not be interpreted as evidence of a supplement-specific anti-inflammatory effect. Although IL-8 and VEGF did not show statistically significant longitudinal changes, their descriptive trajectories were retained as exploratory observations. CRP is a clinically accessible marker reflecting systemic inflammatory activity and has been associated with poor prognosis and treatment outcomes in colorectal cancer patients [21,22,23]. Notably, patients with higher baseline CRP concentrations exhibited greater reductions during follow-up. This aspect may be particularly relevant in older adults, in whom immunosenescence and inflammaging contribute to persistent low-grade inflammation and may influence cancer progression. However, this association should be interpreted cautiously, because correlations between a baseline value and its subsequent change may be influenced by mathematical coupling and regression to the mean; therefore, it should not be considered evidence that patients with higher baseline inflammation necessarily derive greater biological benefit from supplementation [24]. Although the present study was not designed to investigate the molecular mechanisms underlying the observed changes, the observed longitudinal biomarker changes can be discussed in the context of current concepts of inflammaging, in which age-related DNA damage promotes persistent activation of cGAS–STING- and NF-κB-dependent inflammatory signaling [25,26,27]. The supplementation with an anthocyanin-rich preparation was generally well-tolerated despite the relatively high tablet burden.

The observed reduction in CRP concentrations is of particular clinical interest, as systemic inflammation is increasingly recognized as a key component of colorectal cancer biology. Elevated CRP concentrations have been associated with greater tumour burden, poorer nutritional status, reduced treatment tolerance, and unfavorable clinical outcomes in patients with colorectal cancer [21,22,23]. Among participants with paired CRP measurements at baseline and Week 12, CRP concentrations decreased in the majority of participants, with more than half showing a reduction of at least 50% after three months of follow-up. This post hoc threshold was used solely as a descriptive exploratory category and should not be interpreted as a prespecified or clinically validated response criterion. This finding indicates that the observed CRP reduction was not confined to a small subgroup of participants. The magnitude of this change should nevertheless be interpreted cautiously. Meta-analyses of randomized anthocyanin interventions have generally reported substantially smaller average reductions in CRP and considerable heterogeneity between studies [12,13]. In addition, supplementation in the present study lasted only 21 days, and the Week 12 assessment took place approximately nine weeks after its completion, during ongoing oncological treatment. Consequently, the CRP trajectory is noteworthy, but its full magnitude and persistence cannot be attributed to anthocyanin supplementation alone. The concomitant longitudinal changes in CRP, IL-6, and TNF-α Net MFI are biologically compatible with the known relationships among these inflammatory pathways. Hepatic CRP synthesis is primarily driven by IL-6 and may be further influenced by TNF-α- and IL-1-mediated signaling [23]. From a clinical perspective, these observations are particularly relevant because CRP constitutes a central component of several validated prognostic indices in colorectal cancer, including the modified Glasgow Prognostic Score (mGPS), the CRP-to-albumin ratio (CAR), and other inflammation-based prognostic models [21,28]. Although the present study was not designed to evaluate survival or treatment-related outcomes, the observed CRP trajectory warrants further investigation in controlled studies. Whether changes in systemic inflammatory biomarkers associated with nutritional interventions translate into clinically meaningful outcomes remains to be determined.

Human interventional evidence on anthocyanin-rich preparations in colorectal neoplasia remains limited. In a presurgical pilot study, Thomasset et al. administered a standardized bilberry anthocyanin extract to 25 patients with CRC for seven days and demonstrated measurable anthocyanins in colorectal tissue together with a modest reduction in tumour cell proliferation [15]. Wang et al. subsequently reported modulation of tissue biomarkers related to proliferation, apoptosis, Wnt signaling, and angiogenesis after administration of freeze-dried black raspberry powder to patients with CRC, whereas an accompanying analysis by Mentor-Marcel et al. showed a less uniform systemic cytokine response [14,16]. More recently, a randomized presurgical study in patients with colorectal adenomas found no significant between-group differences in changes in circulating inflammatory biomarkers after combined anthocyanin and curcumin supplementation [17]. Thus, the present study extends the available clinical evidence from predominantly short presurgical or prevention-oriented settings to patients subsequently undergoing routine CRC treatment, although this setting also introduces treatment-related confounding. An additional consideration is that anthocyanin exposure in supplementation studies may differ substantially from habitual dietary intake. In the EPIC population, estimated mean dietary anthocyanidin intake across European centres was generally in the range of approximately 20–65 mg/day, whereas the present intervention provided approximately 600–700 mg of anthocyanins per day [29]. These values are not directly analytically equivalent, but they illustrate the substantially higher anthocyanin exposure provided by the study preparation. Importantly, differences between supplementation and consumption of anthocyanin-rich foods cannot be attributed to dose alone. Whole foods, freeze-dried fruit preparations, and purified extracts differ in food matrix, accompanying polyphenols and other phytochemicals, and potentially in anthocyanin bioavailability [30]. Therefore, the findings of the present study should not be directly extrapolated to habitual consumption of anthocyanin-rich foods.

The longitudinal changes observed in CRP, IL-1α Net MFI, TNF-α Net MFI, and IL-6 involve biomarkers that participate in interconnected inflammatory pathways. This pattern is biologically plausible, as these biomarkers are regulated through overlapping signaling networks centered on the nuclear factor kappa B (NF-κB) pathway, which plays a pivotal role in orchestrating inflammatory responses. Activation of NF-κB promotes transcription of numerous pro-inflammatory genes, resulting in increased production of cytokines and inflammatory mediators, including TNF-α, IL-1α, IL-1β, IL-6, IL-8, and CRP. Experimental studies have shown that anthocyanins attenuate inflammatory signaling by inhibiting NF-κB activation, suppressing Toll-like receptor (TLR)-mediated signaling, and modulating MAPK pathways, leading to reduced expression of pro-inflammatory cytokines and inflammatory enzymes such as COX-2 and inducible nitric oxide synthase (iNOS) [10,11]. Importantly, these mechanistic effects have also been demonstrated in humans, with anthocyanin supplementation shown to inhibit NF-κB activation in monocytes and reduce circulating concentrations of pro-inflammatory mediators [31]. In addition, anthocyanins reduce reactive oxygen species (ROS) generation, directly scavenge free radicals, chelate transition metal ions involved in oxidative reactions, and enhance endogenous antioxidant defense systems, thereby attenuating oxidative stress and amplifying their anti-inflammatory activity [10,32]. Owing to these pleiotropic mechanisms, anthocyanins may modulate several components of the inflammatory cascade simultaneously rather than targeting a single inflammatory mediator [33]. The longitudinal changes observed in the present study are biologically compatible with these proposed mechanisms; however, the present uncontrolled study cannot determine whether the observed biomarker changes resulted from the supplementation [9,34]. It should be noted, however, that IL-1α and TNF-α were evaluated as Net MFI because their signals were below the reliable quantitative range of the assay; these findings therefore represent relative changes in signal intensity rather than quantified changes in circulating cytokine concentrations.

Persistent low-grade inflammation is a hallmark of colorectal cancer and plays a central role in both tumor initiation and progression [2,5]. Chronic inflammatory signaling promotes oxidative stress, DNA damage, epithelial dysfunction, and activation of pro-tumorigenic pathways, particularly those mediated by NF-κB. The causal relationship between chronic inflammation and colorectal carcinogenesis is best illustrated by inflammatory bowel disease, in which prolonged intestinal inflammation markedly increases the risk of colitis-associated colorectal cancer through progressive accumulation of genetic alterations within chronically inflamed colonic tissue [6]. Beyond tumor initiation, persistent inflammation remodels the tumor microenvironment, suppresses anti-tumor immunity, and promotes cancer cell survival, proliferation, angiogenesis, and immune evasion [2,35,36]. In this context, the longitudinal changes observed in selected inflammatory biomarkers warrant further investigation. However, because the study was not designed to assess oncological outcomes and did not include a longitudinal CRC control group, no clinical benefit or supplement-specific anti-inflammatory effect can be inferred from these findings.

An important finding of the present study was the generally good tolerability of the anthocyanin-rich preparation despite the relatively high daily tablet burden. Most participants reported no supplementation-related complaints, and the reported problems were predominantly minor, with the presence of small fruit seeds in the lyophilized formulation being the most frequent. In an exploratory analysis, older age was associated with poorer reported tolerability, whereas ECOG performance status, BMI, and nutritional status assessed using MNA and MNA-SF were not significantly associated with tolerability. Because the less tolerant categories included relatively few participants, the association between age and tolerability should be regarded as exploratory and interpreted cautiously. This observation may be particularly relevant in older patients with colorectal cancer, in whom treatment burden, comorbidities, and age-related functional changes may affect adherence to nutritional interventions. Participants also generally reported favorable palatability and practical feasibility of incorporating the preparation into commonly consumed foods and beverages. Taken together, these findings suggest that short-term supplementation with the anthocyanin-rich preparation was feasible and acceptable in this population, although simplification of the formulation and reduction of the daily tablet burden could further improve its practical use.

The present study has several strengths that should be considered when interpreting the findings. First, its prospective interventional design focused specifically on older patients with colorectal cancer, a population that remains underrepresented in nutritional intervention studies. Second, the simultaneous assessment of classical inflammatory markers together with multiple pro- and anti-inflammatory cytokines provided a comprehensive characterization of the systemic inflammatory response. Third, repeated assessments at baseline, 4 weeks, and 12 weeks enabled longitudinal evaluation of inflammatory responses over time. In addition, the study used an anthocyanin-rich freeze-dried whole-fruit preparation developed specifically for this intervention, with previously published phytochemical analyses of the selected fruit materials [20]. Another strength is that the study was conducted under routine clinical conditions, supporting the relevance of the findings to real-world oncological practice.

Several limitations of this study should be acknowledged. First, the study was conducted at a single center with a relatively small sample size, which may limit the generalizability of the findings. In addition, although the study was originally designed for patients aged ≥65 years, six participants aged 61–64 years were enrolled, representing a deviation from the prespecified age criterion and slightly broadening the intended age range of the study population. Although the prospective design and repeated longitudinal assessments enabled characterization of biomarker trajectories over time, the modest sample size limited the precision of the estimates and the ability to detect smaller longitudinal changes or associations. Another important limitation is the clinical heterogeneity of the study population. Participants represented different stages of colorectal cancer (stages II–IV) and, consequently, underwent different treatment strategies according to routine clinical practice. Depending on disease stage and treatment qualification, patients received surgery alone, neoadjuvant chemoradiotherapy followed by surgery, or systemic therapy for metastatic disease. Because systemic inflammation may be influenced by tumour burden as well as by the type and intensity of oncological treatment, these factors may have contributed to the variability of the measured biomarkers. Importantly, the non-cancer comparator group was included only for baseline comparisons and was not followed longitudinally. Therefore, the study did not include a longitudinal control group of patients with colorectal cancer receiving standard oncological treatment without anthocyanin supplementation. Because follow-up assessment was performed during ongoing oncological management, changes in systemic inflammation cannot be attributed solely to anthocyanin supplementation. In addition, because the intervention consisted of freeze-dried whole-fruit material containing other biologically active phytochemicals in addition to anthocyanins, any observed biomarker changes cannot be attributed specifically to anthocyanins. Importantly, the Week 4 assessment was performed before initiation of oncological treatment, whereas the Week 12 assessment was performed during standard oncological treatment. Consequently, the longer-term biomarker trajectories between Week 4 and Week 12 may have been influenced by surgery, chemoradiotherapy, or systemic treatment and cannot be attributed to the preceding anthocyanin supplementation. Recent longitudinal evidence in patients with colorectal cancer has also demonstrated that inflammatory biomarker trajectories may differ according to oncological treatment modality, further supporting the need for caution when interpreting the Week 12 findings [37]. In addition, intercurrent infections, postoperative complications, hospitalizations, and concomitant medications capable of modifying inflammatory biomarkers, including antibiotics, corticosteroids, and non-steroidal anti-inflammatory drugs, were not systematically recorded or controlled for and may have contributed to the observed biomarker variability. Owing to the relatively small sample size, subgroup analyses according to disease stage or treatment modality were not feasible and should be addressed in future studies. The intervention period consisted of 21 days of supplementation, whereas participants were followed for 12 weeks. Therefore, the present study cannot determine the optimal dose or duration of supplementation or whether any of the observed biomarker changes persist independently of concomitant oncological treatment. In addition, missing repeated measurements resulted in complete-case longitudinal analyses for individual biomarkers, and the assessment of a broad biomarker panel raises the possibility of multiplicity-related findings; these issues should be considered when interpreting individual statistical associations. Moreover, dietary intake was not standardized or systematically monitored; therefore, potential changes in the consumption of anthocyanin- and polyphenol-rich foods or other dietary components may have contributed to variability in the measured biomarkers. Finally, although longitudinal changes were observed in several inflammatory biomarkers, the present study was not designed to evaluate hard clinical outcomes such as treatment response, postoperative complications, recurrence-free survival, or overall survival. Whether these biomarker changes have clinical relevance requires evaluation in adequately powered controlled trials.

## 5. Conclusions

Longitudinal changes in systemic inflammatory biomarkers, including a decrease in CRP and changes in several inflammatory markers, were observed during follow-up in patients who had received a 21-day course of anthocyanin-rich berry supplementation before initiation of standard oncological treatment. The supplementation course was generally well-tolerated. However, because the study lacked a longitudinal CRC control group and participants underwent heterogeneous oncological treatment, these biomarker changes cannot be attributed specifically to the supplementation. The findings should therefore be considered exploratory and hypothesis-generating rather than evidence of a treatment effect. Nevertheless, they provide a clinically relevant rationale for further evaluation of the potential of anthocyanin-rich berry supplementation to influence systemic inflammatory biomarkers in this setting. Further randomized, prospectively registered studies with predefined endpoints and appropriate control for concomitant oncological treatment are warranted.

## Figures and Tables

**Table 1:** Baseline characteristics of the study population.

Parameter		Study Group (*N* = 50)	Baseline Comparator Group (*N* = 20)	*p*
Age [years]	Mean (SD)	69.68 (5.73)	69.3 (6.39)	*p* = 0.907
	Median (quartiles)	68 (65–73)	68.5 (65–73.25)	
	Range	61–83	59–83	
	*n*	50	20	
Sex	Female	19 (38%)	16 (80%)	*p* = 0.004 *
	Male	31 (62%)	4 (20%)	
Baseline ECOG	ECOG 0	24 (48%)	12 (60%)	*p* = 0.307
	ECOG 1	25 (50%)	7 (35%)	
	ECOG 2	1 (2%)	1 (5%)	
Baseline BMI [kg/m^2^]	Mean (SD)	26.47 (4.84)	27.52 (5.26)	*p* = 0.608
	Median (quartiles)	25.81 (23.15–30.45)	26.32 (24.13–29.56)	
	Range	16.56–37.22	20.08–40.86	
	*n*	50	20	

*p*—Qualitative variables: chi-squared or Fisher’s exact test. Quantitative variables: Mann–Whitney test. * statistically significant (*p* < 0.05).

**Table 2:** Comparison of baseline demographic, clinical, and laboratory characteristics between the study and baseline comparator group.

Parameter	Group	*N*	Mean	SD	Median	Min	Max	Q1	Q3	*p*
Baseline CRP [mg/L]	Study group	50	29.85	30.34	20.72	0.28	122.52	3.11	49.29	*p* = 0.007 *
	Comparator group	20	12.41	24.77	3.26	0.6	99.58	0.6	8.32	
Baseline CEA [ng/mL]	Study group	50	230.76	853.65	4.73	0.91	5610	2.04	42.59	*p* < 0.001 *
	Comparator group	17	1.81	0.84	1.7	0.74	3.45	0.93	2.24	
Baseline TP [g/L]	Study group	45	65.07	8.46	65.09	43.5	79.7	61.8	71.5	*p* = 0.161
	Comparator group	20	68.38	5.58	70	55	79.7	67.25	71.05	
Baseline albumin [g/L]	Study group	45	37.31	6.44	37	22	50	32	42	*p* = 0.235
	Comparator group	20	39.15	5.93	38	30	48	35	45	
Baseline IL-1α [Net MFI]	Study group	50	2.29	1.38	2.16	0.22	7.16	1.57	2.65	*p* = 0.416
	Comparator group	20	1.93	1.06	2.02	0	3.74	1.23	2.64	
Baseline IL-1β [Net MFI]	Study group	50	2.41	1.66	2.46	0	5.85	0.85	3.64	*p* = 0.01 *
	Comparator group	20	1.28	1.09	1.31	0	4.41	0.6	1.71	
Baseline IL-10 [pg/mL]	Study group	50	3.11	4.65	1.59	0.16	31.01	0.88	3.97	*p* = 0.183
	Comparator group	20	3.44	2.55	3.04	0.43	8.86	1.21	5.46	
Baseline VEGF [pg/mL]	Study group	50	122.29	141.57	75.16	11.91	852.99	43.23	142.28	*p* = 0.511
	Comparator group	20	123.85	95.99	104.67	14.44	356.09	50.72	164.72	
Baseline TNF-α [Net MFI]	Study group	50	10.77	5.74	10.25	0.7	28.22	7	14.22	*p* = 0.649
	Comparator group	20	10.51	6.78	9.14	1.65	29.86	6.42	14.02	
Baseline IL-6 [pg/mL]	Study group	50	3.32	8.05	0.87	0	55.32	0.17	3.17	*p* = 0.88
	Comparator group	20	3.4	5.67	2.74	0	25.81	0	4.3	
Baseline IL-8 [pg/mL]	Study group	50	33.84	57.11	12.48	2.73	302.26	7.17	25.31	*p* = 0.73
	Comparator group	20	18.48	16.39	11.25	4.42	66.63	8.3	24.27	
Baseline EGF [pg/mL]	Study group	50	168.26	168.39	107	3.63	806.76	65.56	210.56	*p* = 0.692
	Comparator group	20	195.65	177.3	173.42	4.67	546.99	46.8	273.03	

*p*—Mann–Whitney test, SD—standard deviation, Q1—lower quartile, Q3—upper quartile. * statistically significant (*p* < 0.05).

**Table 3:** Longitudinal changes in anthropometric measurements, biochemical parameters, inflammatory biomarkers, angiogenic factors, and tumor markers in the supplementation group at baseline, Week 4, and Week 12.

Parameter	Measurement	*N*	Mean	SD	Median	Min	Max	Q1	Q3	*p*
Weight [kg]	Baseline	46	73.43	14.77	70.00	49.00	114.00	65.25	83.75	*p* = 0.042 * 3M > 1M
	Week 4	46	72.88	14.68	70.00	47.00	121.00	64.00	78.75	
	Week 12	46	74.48	14.55	71.50	49.00	124.00	66.00	83.50	
BMI [kg/m^2^]	Baseline	46	26.54	4.83	25.48	16.56	37.22	23.15	30.45	*p* = 0.042 * 3M > 1M
	Week 4	46	26.31	4.58	25.62	15.89	39.51	23.05	30.01	
	Week 12	46	26.90	4.57	26.39	16.56	40.49	23.87	29.87	
CRP [mg/L]	Baseline	43	28.19	28.35	20.44	0.28	92.33	2.84	46.38	*p* < 0.001 * B > 1M > 3M
	Week 4	43	19.87	27.37	5.22	0.50	100.05	2.03	36.02	
	Week 12	43	14.97	31.09	3.00	0.60	188.11	1.27	15.84	
CEA [ng/mL]	Baseline	43	236.88	906.98	5.72	0.91	5610.00	2.02	41.26	*p* = 0.073
	Week 4	43	174.64	480.24	3.18	0.69	2043.00	1.92	28.66	
	Week 12	43	31.40	82.79	2.83	0.61	379.00	1.67	6.95	
TP [g/L]	Baseline	37	65.19	8.59	65.00	43.50	79.70	61.80	71.90	*p* = 0.13
	Week 4	37	66.62	6.73	67.10	50.00	80.00	62.30	72.00	
	Week 12	37	66.00	7.47	68.00	43.40	79.00	65.00	70.00	
Albumin [g/L]	Baseline	37	38.24	6.54	40.00	22.00	50.00	33.00	43.00	*p* = 0.674
	Week 4	37	37.78	6.33	38.00	21.00	49.00	34.00	42.00	
	Week 12	37	37.97	6.03	38.00	21.00	49.00	35.00	42.00	
IL-1α [Net MFI]	Baseline	39	2.19	1.36	2.13	0.22	7.16	1.54	2.48	*p* = 0.003 * B, 1M > 3M
	Week 4	39	1.86	0.87	1.80	0.00	3.76	1.33	2.32	
	Week 12	39	1.43	1.00	1.40	0.00	5.35	0.62	1.75	
IL-1β [Net MFI]	Baseline	39	2.61	1.59	2.55	0.00	5.85	1.40	3.60	*p* = 0.264
	Week 4	39	2.43	1.55	2.21	0.00	6.00	1.15	3.62	
	Week 12	39	2.00	1.47	1.56	0.00	5.27	0.86	3.12	
IL-10 [pg/mL]	Baseline	39	2.39	2.35	1.52	0.27	10.65	0.81	3.58	*p* = 0.624
	Week 4	39	2.72	2.37	1.80	0.00	9.46	1.00	3.77	
	Week 12	39	2.11	1.91	1.66	0.00	10.14	0.86	2.74	
VEGF [pg/mL]	Baseline	39	99.07	91.26	65.42	11.91	421.21	36.22	133.17	*p* = 0.143
	Week 4	39	94.01	87.37	62.63	9.98	400.53	34.32	118.15	
	Week 12	39	74.92	66.11	50.22	11.59	343.20	35.92	94.26	
TNF-α [Net MFI]	Baseline	39	10.74	5.71	9.26	2.20	28.22	6.86	14.20	*p* < 0.001 * B > 3M
	Week 4	39	9.63	5.92	8.29	1.22	26.23	5.90	12.52	
	Week 12	39	8.07	5.29	8.26	0.00	25.25	4.76	10.96	
IL-6 [pg/mL]	Baseline	39	2.09	2.90	0.87	0.00	15.41	0.17	3.05	*p* = 0.038 * 1M > 3M
	Week 4	39	2.46	4.35	0.87	0.00	25.60	0.17	3.09	
	Week 12	39	1.94	3.44	0.17	0.00	12.84	0.00	2.30	
IL-8 [pg/mL]	Baseline	39	31.03	55.36	11.10	2.73	302.26	6.61	23.08	*p* = 0.489
	Week 4	39	26.70	49.98	9.57	2.31	263.21	6.49	17.23	
	Week 12	39	29.12	50.99	15.04	2.31	294.88	7.86	20.87	
EGF [pg/mL]	Baseline	38	158.62	156.87	107.00	7.61	806.76	66.33	191.13	*p* = 0.442
	Week 4	38	187.03	170.69	142.82	6.63	767.52	45.25	314.57	
	Week 12	38	119.38	113.65	80.25	1.98	459.93	22.79	196.84	

*p*—Friedman test + post hoc analysis (Wilcoxon paired tests with Bonferroni correction). * statistically significant (*p* < 0.05).

**Table 4:** Magnitude of changes in *C*-reactive protein (CRP) concentrations after 3 months of follow-up in the supplementation group.

CRP Decrease After 3 Months [mg/L]								
*N*	Missing	Mean	SD	Median	Min	Max	Q1	Q3
43	7	13.22	34.53	11.9	−114.12	86	0	34.3

**Table 5:** Association between baseline *C*-reactive protein (CRP) concentrations and the magnitude of CRP reduction after 3 months of follow-up.

Variables	Spearman’s Correlation Coefficient	*p*
Baseline CRP & CRP decrease after 3 months	0.8	*p* < 0.001

**Table 6:** Magnitude of changes in IL-8 concentrations after 3 months of follow-up in the supplementation group.

IL-8 Decrease After 3 Months [pg/mL]								
*N*	Missing	Mean	SD	Median	Min	Max	Q1	Q3
42	8	1.8	53.35	0.38	−129.11	240.26	−8	7.04

SD—standard deviation, Q1—lower quartile, Q3—upper quartile.

**Table 7:** Association between baseline IL-8 concentrations and the magnitude of IL-8 reduction after 3 months of follow-up in the supplementation group.

Variables	Spearman’s Correlation Coefficient	*p*
Baseline IL-8 & IL-8 decrease after 3 months	0.509	*p* = 0.001

**Table 8:** Magnitude of changes in VEGF concentrations after 3 months of follow-up in the supplementation group.

VEGF Decrease After 3 Months [pg/mL]								
*N*	Missing	Mean	SD	Median	Min	Max	Q1	Q3
42	8	24.77	75.37	10.49	−137.26	309.29	−4.79	30.27

SD—standard deviation, Q1—lower quartile, Q3—upper quartile.

**Table 9:** Association between baseline VEGF concentrations and the magnitude of VEGF reduction after 3 months of follow-up in the supplementation group.

Variables	Spearman’s Correlation Coefficient	*p*
Baseline VEGF & VEGF decrease after 3 months	0.609	*p* < 0.001

**Table 10:** Association of baseline characteristics with reported supplementation tolerability.

Parameter		Good-A (*N* = 37)	Moderate-B (*N* = 4)	Medium/Low-C (*N* = 6)	*p*
Age [years]	Mean (SD)	68.54 (5.43)	72.25 (4.65)	76.33 (4.76)	*p* = 0.005 * C > A
	Median (quartiles)	67 (65–72)	71.5 (68.75–75)	77 (73.75–79.5)	
	Range	61–83	68–78	69–82	
	*n*	37	4	6	
Baseline BMI [kg/m^2^]	Mean (SD)	26.63 (5.1)	30.5 (2.86)	24.41 (2.07)	*p* = 0.072
	Median (quartiles)	26.03 (23.23–30.46)	31.4 (29.67–32.23)	24.06 (23.07–25.03)	
	Range	16.56–37.22	26.4–32.79	22.31–27.97	
	*n*	37	4	6	
MNA	Mean (SD)	22.54 (3.57)	23.62 (4.03)	19.83 (5.56)	*p* = 0.459
	Median (quartiles)	23 (21–24)	23.5 (20.62–26.5)	20.75 (18.5–23.75)	
	Range	12.5–28	19.5–28	10–25	
	*n*	37	4	6	
MNA-SF	Mean (SD)	10.19 (2.61)	10.5 (3.51)	9.17 (2.79)	*p* = 0.604
	Median (quartiles)	10 (8–12)	10.5 (7.75–13.25)	8.5 (7.25–11.25)	
	Range	4–15	7–14	6–13	
	*n*	37	4	6	
Baseline ECOG	ECOG 0	21 (56.76%)	2 (50.00%)	1 (16.67%)	*p* = 0.24
	ECOG 1	16 (43.24%)	2 (50.00%)	5 (83.33%)	
	ECOG 2	0 (0.00%)	0 (0.00%)	0 (0.00%)	
	ECOG 3	0 (0.00%)	0 (0.00%)	0 (0.00%)	

*p*—Qualitative variables: chi-squared or Fisher’s exact test. Quantitative variables: Kruskal–Wallis test + post hoc analysis (Dunn test). * statistically significant (*p* < 0.05).

**Table 11:** Reported complaints related to anthocyanin supplementation.

Problems	*n*	%
Pits	5	10%
Pits + anorexia	2	4%
Sour taste and pits	1	2%
Too many tablets	2	4%
None	38	76%
Unknown	2	4%

Note: Percentages are calculated using the total intervention cohort (*N* = 50); complaint-specific information was available for 48 participants, while data were unavailable for 2 participants.

## Data Availability

The data supporting the findings of this study are available from the corresponding author upon reasonable request. The data are not publicly available due to privacy and ethical considerations.
